# Dopamine activity on the perceptual salience for recognition memory

**DOI:** 10.3389/fnbeh.2022.963739

**Published:** 2022-07-25

**Authors:** Daniel Osorio-Gómez, Kioko Guzmán-Ramos, Federico Bermúdez-Rattoni

**Affiliations:** ^1^División de Neurociencias, Instituto de Fisiología Celular, Universidad Nacional Autónoma de México, Circuito Exterior, Ciudad Universitaria, Mexico, Mexico; ^2^Departamento de Ciencias de la Salud, División de Ciencias Biológicas y de la Salud, Universidad Autónoma Metropolitana, Unidad Lerma, Estado de México, Mexico

**Keywords:** novelty, catecholamines, saliency, contextual, perception

## Abstract

To survive, animals must recognize relevant stimuli and distinguish them from inconspicuous information. Usually, the properties of the stimuli, such as intensity, duration, frequency, and novelty, among others, determine the salience of the stimulus. However, previously learned experiences also facilitate the perception and processing of information to establish their salience. Here, we propose “perceptual salience” to define how memory mediates the integration of inconspicuous stimuli into a relevant memory trace without apparently altering the recognition of the physical attributes or valence, enabling the detection of stimuli changes in future encounters. The sense of familiarity is essential for successful recognition memory; in general, familiarization allows the transition of labeling a stimulus from the novel (salient) to the familiar (non-salient). The novel object recognition (NOR) and object location recognition (OLRM) memory paradigms represent experimental models of recognition memory that allow us to study the neurobiological mechanisms involved in episodic memory. The catecholaminergic system has been of vital interest due to its role in several aspects of recognition memory. This review will discuss the evidence that indicates changes in dopaminergic activity during exposure to novel objects or places, promoting the consolidation and persistence of memory. We will discuss the relationship between dopaminergic activity and perceptual salience of stimuli enabling learning and consolidation processes necessary for the novel-familiar transition. Finally, we will describe the effect of dopaminergic deregulation observed in some pathologies and its impact on recognition memory.

## Introduction

Organisms are continuously exposed to several stimuli and events in their environment across their lifespans. Nevertheless, individuals must efficiently and effectively guide their behavior according to the perceived relevant stimuli. The continuous processing of incoming information demands considerable cognitive effort. Therefore, selecting, filtering, and processing information is essential to preserve proper cognitive function. In this regard, the relevant information is processed with less cognitive interference, characterized by the competition of information in eliciting cognitive processes (Grachev et al., 2001) compared to neutral or inconspicuous stimuli. Selecting relevant information from the environment is easily achieved when these stimuli are intrinsically salient due to their physical properties. Thus, salience refers to the phenomenon by which a stimulus highlights or is set apart from the environment (Uddin, 2015). Generally, a salient stimulus attracts attentional resources bottom-up to facilitate information processing (Santangelo, 2015), where the stimulation drives cognitive processes that contrasts with the surroundings. Bottom-up processing states that the stimuli’s physical attributes originate from sensory information facilitating salience and perception (Riener, 2019). In this regard, perceptual processing determines the attention and cognition required for a proper behavioral reaction to the stimuli in the environment (Goldstein and Cacciamani, 2021). However, there is evidence suggesting that the physical properties of the stimuli are not the only factors that drive information processing. Top-down processing, also called knowledge-based processing, refers to the “internal” factors of the observer acquired by previous experiences (Awh et al., 2012). Top-down processing is driven by cognition, starting with memory and expectations that affect salience and perception (Riener, 2019). Therefore, the brain hierarchizes salient information according to the physical stimuli properties and the organism’s experience, facilitating perception of the stimuli.

Current evidence indicates that perception and memory interact to direct and control driving attentional and cognitive processes actions (for a review, Heurley and Ferrier, 2015). A salient stimulus is more prone to be integrated into memory traces than non-salient stimuli. Even though many stimuli appear to be non-salient due to their intrinsic low-intensity properties; they can become salient based on their meaning, consequences, or relationship with other stimuli in the environment (Santangelo, 2015). Therefore, previous experiences modulate stimuli recognition by comparing them with stored information and adjusting salience processing (Riener, 2019). Here, we propose the term perceptual salience referring to how memory modulates the integration of inconspicuous stimuli into a relevant memory without enhancing the initial sensory perception.

Memory is a fundamental adaptative mechanism that promotes organisms’ survival by identifying relevant environmental changes. Across their lifespan, animals experience several episodes associated with important information about food, shelter, and danger, among others. Thus, individuals need to recall those specific events and appropriately modify their behavior to survive. The encoding, integration, and retrieval of experienced information is defined as memory (Squire, 2009). Overall, memory formation requires acquiring information by learning events during exposure to stimuli. Then, memories are integrated into long-lasting traces through chemical and structural modification *via* protein synthesis (McGaugh, 2000; Bisaz et al., 2014). When necessary, internal and external cues promote selection, reactivation, and assessment of information, modulating the behavioral outcomes, a process called memory retrieval (Ben-Yakov et al., 2015; Frankland et al., 2019). During memory retrieval, memories undergo a consolidation-like process called reconsolidation, where memory updating may occur (Nader et al., 2000; Sara, 2000; Lee et al., 2017; Rodriguez-Ortiz and Bermúdez-Rattoni, 2017). Memory engages distinct neural circuits or systems accordingly to the type of information, inducing cellular and molecular changes that support memory maintenance (Nadel and Hardt, 2011).

According to the awareness during retrieval, memory systems have been classified into declarative and non-declarative. Non-declarative memories are characterized by integrating information associated with habits, motor learning, and associative learning. The most common example is the information accessed without conscious recall. Conversely, declarative memories are recalled consciously and are related to facts (semantic memory) and events (episodic memory; Squire, 2009; Nadel and Hardt, 2011). Specifically, episodic memory integrates “where,” “what,” and “when” an event happened into a spatiotemporal context (Tulving, 2002). Therefore, an essential aspect of episodic memory is the judgment of whether a recent experience, including subject, location, or event, has been previously experienced or encountered. Episodic memory integrates information related to environmental changes, facilitating the identification of different information modalities, including faces, places, sounds, objects, or changes in the context. The integrated information allows the discrimination of novel events from familiar ones. Thus, recognition memory involves familiarization by acquiring, consolidating, retrieving, and updating experienced events in a space-time frame (Squire and Zola, 1996; Tulving, 2002; Balderas et al., 2015; Morici et al., 2015).

In general, recognition memory incorporates two differential processes: Recollection and Familiarity (Brown and Aggleton, 2001; Merkow et al., 2015). Familiarity is the ability to judge whether a particular stimulus or event has already been experienced (Mandler, 1980). In contrast, recollection retrieves the stimuli or events’ characteristics (qualitative dimension; Evans and Wilding, 2012). Thus, exposure to novelty (salient stimulus) triggers a maximum behavioral response that is progressively reduced during subsequent presentations (familiar, non-salient stimulus). The novelty transitions to familiarity are gradual shifts caused by learning (Henson and Gagnepain, 2010) and neuronal plasticity changes (Lisman et al., 2011). Recollection of contextual events and their behavioral responses occur by activating several brain regions (Kafkas and Montaldi, 2014), like the entorhinal cortex (Knierim, 2015), hippocampus (Barker and Warburton, 2011), and the prefrontal cortex (Akirav and Maroun, 2006). In comparison, the items’ familiarity variations rely on parahippocampal (perirhinal, entorhinal, and postrhinal; Brown and Aggleton, 2001; Yonelinas, 2002; Evans and Wilding, 2012; Merkow et al., 2015) and insular (Bermudez-Rattoni et al., 2005; Balderas et al., 2008) cortices of the brain. Within these structures, changes in the neurotransmitters involved in the transition from novelty to familiarity include elevation in acetylcholine, noradrenaline, and dopamine which gradually diminish after the consecutive exposure to the stimulus (Miranda et al., 2000; Osorio-Gómez et al., 2016, 2017; Rodríguez-García and Miranda, 2016). The activation of the same neurotransmission systems occurs in different areas of the brain depending on memory recollection of events or stimuli recognition. Even though many neurotransmitters are involved in recognition memory, the catecholaminergic system is of particular interest due to its modulatory effect on synaptic plasticity and memory processes that might impact perceptual salience (Jay, 2003; Lisman et al., 2011; Takeuchi et al., 2016; Yang et al., 2017).

Finally, we will review how cognitive impairments are directly related to dopaminergic dysfunctions, impacting recognition memory in pathologies such as Alzheimer’s disease (Guzmán-Ramos et al., 2012; Moreno-Castilla et al., 2017), schizophrenia (Brisch et al., 2014), and Parkinson’s disease (Aarsland, 2016). Abnormal functioning in several brain regions has been related to recognition memory detriments observed during spontaneous exploration tasks in the animal model used to study these processes.

## Spontaneous Object Exploration

**Figure 1:**
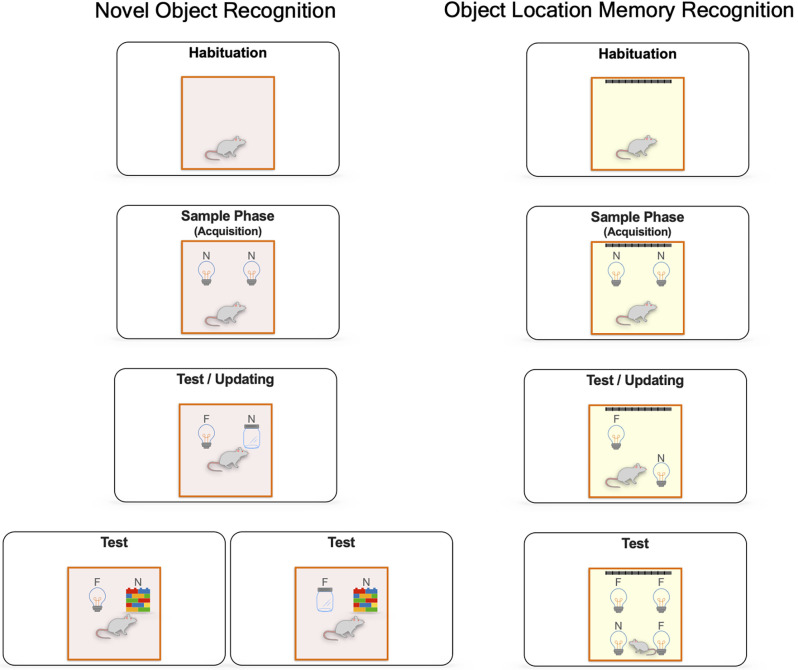
General procedures for spontaneous object exploration. Novel object recognition (NOR) and object location recognition memory (OLRM) paradigms include different sessions: a handling and habituation period to an empty open field to reduce stress and promote the exploratory activity; a sample phase, where the animal is given a period to freely explore two identical objects. During the test session, short-term or long-term assessment of recognition memory is determined by the length of the retention interval, in this session the animal freely explores a different novel object (jar) in the case of NOR or the displacement of an object to a novel location in the case of OLRM. Finally, recognition memory updating is evaluated if a new third object is presented (wall of brick toys) in a NOR task or when a new displaced position is presented in an OLRM task. In general, animals that remember the familiar object of the familiar spatial configuration will spend more time exploring the novel object or the novel spatial configuration Novel (N) and Familiar (F).

Spontaneous novel object recognition tasks are widely used to assess long-term recognition memory’s neurobiological mechanisms. Novel object recognition (NOR) and object location recognition memory (OLRM) are the most common behavioral paradigms employed to determine the processes of acquisition, consolidation, retrieval, and updating of recognition memory (see Figure 1). NOR and OLRM are simple tasks based on the rodents’ innate preference to explore novel stimuli than familiar ones (Ennaceur and Delacour, 1988; Chan et al., 2018). Both paradigms consist of at least three sessions: a handling and habituation period to an empty open field. Then, a sample phase (acquisition session), where animals explore novel objects for first-time. Finally, a test session (retrieval) where animals discriminate and identify a novel object in the case of NOR or the displaced one in the case of OLRM (Ameen-Ali et al., 2015; Chan et al., 2018). In addition, novel information can be integrated during retrieval and updating recognition memory (Balderas et al., 2015; Kwapis et al., 2020; Wright et al., 2020; see Figure 1). These tasks allow us to assess the ability of animals to recognize environmental changes caused by exposure to a novel object or a novel spatial configuration and help determine novel/familiar discrimination and recollection processes.

Several experimental approaches have evaluated the differential participation of several brain structures in the consolidation of recognition memory. Regarding OLRM, the hippocampus is a crucial brain structure in spatial-dependent tasks. Hippocampal lesions impair OLRM (Save et al., 1992; Mumby et al., 2002; Barker and Warburton, 2011). Particularly, CA3 lesions (Lee et al., 2005; Hunsaker et al., 2008) or its pharmacological inactivation (Barbosa et al., 2012) hinder spatial novelty discrimination. In addition, pharmacological inactivation of the CA1 portion impairs OLRM (Assini et al., 2009). Thus, the dorsal CA1 and CA3 portions are related to acquiring, consolidating, and retrieving contextual information (Brown and Aggleton, 2001; Barker and Warburton, 2011; Moreno-Castilla et al., 2017). Concerning NOR, this task depends on the insular cortex (Bermudez-Rattoni et al., 2005; Balderas et al., 2008), the perirhinal cortex (Warburton et al., 2003; Winters et al., 2004; Winters and Bussey, 2005; Balderas et al., 2013b), and the ventromedial prefrontal cortex (Akirav and Maroun, 2006). However, the involvement of the hippocampus in NOR has been controversial (Mumby, 2001; Balderas et al., 2008; Barker and Warburton, 2011; Haettig et al., 2011). Evidence suggests that the functional integrity of the hippocampal activity is required in NOR consolidation (Rossato et al., 2007; Myskiw et al., 2008; Cohen et al., 2013; Furini et al., 2014). For many years it was assumed that hippocampal activity was necessary only when recalling objects in a particular context (Barker and Warburton, 2011). Anisomycin administration into the dorsal hippocampus immediately after the sample phase impairs long-term but not short-term object-in-context recognition memory (Balderas et al., 2008). The object-in-context task is a spontaneous exploration paradigm in which animals spend more time exploring familiar objects within a novel context (salient information) than in a familiar one (non-salient information). However, conflicting results might be explained by differences in experimental approaches (lesions vs. temporal inactivation). The use of a particular behavioral protocol and not merely by the type of information (what vs. where; for review, please see Cohen and Stackman, 2015).

### Catecholaminergic system involvement in NOR and OLMR

The catecholaminergic system is strongly related to cognitive processes and plays a vital role in the modulation of recognition memory (Yang et al., 2017; Titulaer et al., 2021). Exposure to novel stimuli or contextual information induces an elevation in catecholamines and disruption in catecholaminergic activity hinders recognition memory (Guzmán-Ramos et al., 2012; Moreno-Castilla et al., 2017). Dopaminergic and noradrenergic systems regulate neuronal plasticity events related to memory formation and consolidation. So, any alteration in the catecholaminergic system elevates the probability of cognitive impairments, including recognition memory. The main catecholaminergic inputs arise from the locus coeruleus (LC) and the ventral tegmental area (VTA), innervating several brain structures through the mesolimbic and mesocortical pathways (Ungless, 2004; Bromberg-Martin et al., 2010; Takeuchi et al., 2016).

Regarding norepinephrine, the LC supplies projections into several parts of the brain, including the medial temporal lobe (Pudovkina et al., 2001; Aston-Jones and Cohen, 2005; Morilak et al., 2005; Kempadoo et al., 2016). The noradrenergic system plays a critical role in memory consolidation processes by modulating plasticity-related events (McGaugh, 2000, 2013; McGaugh, 2015; Barsegyan et al., 2014). Norepinephrine activates α (α1 and α2) and β (β1, β2, and β3) receptors. Stimulation of α1 receptors increases Ca^2+^ and diacylglycerol intracellular levels, promoting the activation of phospholipase C and the subsequent activation of protein kinase C; this signaling pathway modulates neuronal changes necessary for memory establishment (Perez, 2020). Furthermore, the activation of β receptors promotes adenylate cyclase activity that increases cAMP levels. This augmentation results in the activation of protein kinase A, which ultimately promotes gene expression and memory consolidation (O’Dell et al., 2015).

LC activity increases its firing response after presenting novel objects or contexts (Sara et al., 1994; Vankov et al., 1995; Pudovkina et al., 2001; Wagatsuma et al., 2018). Recent evidence indicates that the LC projects dopaminergic and noradrenergic terminals into the dorsal hippocampus (Kempadoo et al., 2016), enhancing spatial memory consolidation through D1-like receptor activity (Takeuchi et al., 2016). Moreover, the catecholaminergic denervation through the administration of 6-OHDA into the hippocampus interferes with OLRM formation (Moreno-Castilla et al., 2017). A similar lesion in the shell subregion of the nucleus accumbens impairs object location memory (Nelson et al., 2010). Pharmacological activation of the noradrenergic receptors through the systemic administration of epinephrine improves recognition and memory consolidation (Dornelles et al., 2007). Similarly, activation of β receptors within the basolateral amygdala enhances object-in-context recognition memory (Barsegyan et al., 2014), NOR (Chen et al., 2022), and OLRM consolidation (Roozendaal et al., 2007; Song et al., 2021). The noradrenergic system presumably promotes memory consolidation due to arousal effects; since the basolateral amygdala modulates the dorsal hippocampus and insular and prelimbic cortices through the noradrenergic system (Barsegyan et al., 2019; Chen et al., 2022). Likewise, norepinephrine administration into the hippocampus immediately after the sample session promotes NOR persistence (Mello-Carpes et al., 2016). Thus, exposure to novel objects or contextual information enhances memory consolidation through LC modulation, noradrenaline release within the amygdala, and the corelease of norepinephrine and dopamine in the hippocampus. In addition, changes in norepinephrine levels would be associated with relevant stimuli detection, regulating arousal, improving cortical function, and enhancing subsequent cognitive functions such as attention and motivation (Aston-Jones and Cohen, 2005).

The other catecholaminergic neurotransmitter is dopamine, produced in midbrain neurons within the substantia nigra and the VTA (Baik, 2020). Dopamine release from the substantia nigra is mainly involved in controlling motor function and goal-directed behaviors (Grillner et al., 2008). However, dopamine release from the VTA and the LC terminals into the nucleus accumbens, prefrontal cortex, and medial temporal lobe (hippocampus, perirhinal, insular, parahippocampal, and entorhinal cortices) is involved in the formation and maintenance of declarative memories such as recognition memory (de Lima et al., 2011; Kempadoo et al., 2016; Takeuchi et al., 2016; Moreno-Castilla et al., 2017). In general, dopamine is a neuromodulator that modifies functional connectivity during synaptic plasticity (Jay, 2003; Lisman et al., 2011; Otani et al., 2015; Yang et al., 2017). These modifications occur after the activation of metabotropic receptors and the later induction of signaling pathways cascades, promoting the enhancement of neuronal plasticity. Mainly, dopamine activates D1-like (D1 and D5) and D2-like (D2, D3, and D4) receptors; activation of D1 receptors triggers Gs protein inducing an augmentation of cAMP and the subsequent activation of protein kinase A (Undieh, 2010), regulating conductance of NMDA receptors *via* phosphorylation of NR1 and NR2 subunits (Chen et al., 2004; Murphy et al., 2014). Moreover, activation of D1 receptors promotes AMPA (Mangiavacchi and Wolf, 2004; Rozas et al., 2015) and NMDA (Gao and Wolf, 2008; Li et al., 2010) receptors externalization. In the case of D2-like receptors, their activation inhibits adenylate cyclase through Gi proteins, suppressing neurotransmitter release from terminals (Neve et al., 2004).

Dopamine plays an essential role in recognition memory since the dopaminergic neuronal activity is modified by novel and salient stimuli (Ljungberg et al., 1992; Ungless, 2004). The VTA is a dopaminergic nucleus that displays changes in electrical activity associated with the presentation of novel stimuli (Ljungberg et al., 1992; Schultz, 1998; Düzel et al., 2009), increasing dopaminergic levels within the nucleus accumbens (Legault and Wise, 2001; Leonibus et al., 2006), striatum (Ihalainen et al., 1999), the dorsal and ventral hippocampus (Ihalainen et al., 1999; Mello-Carpes et al., 2016; Moreno-Castilla et al., 2017; Hernández-Ramírez et al., 2021; Titulaer et al., 2021), the prefrontal cortex (Feenstra and Botterblom, 1996; Ihalainen et al., 1999; Feenstra et al., 2000), and the insular cortex (Guzmán-Ramos et al., 2010, 2012; Osorio-Gómez et al., 2021). Therefore, the dopaminergic system has been related to novelty detection that triggers recognition memory establishment (Rossato et al., 2013; Otani et al., 2015; Moreno-Castilla et al., 2016; Yang et al., 2017). Object and location recognition memory depends on catecholaminergic activity. Exploring novel objects induces dopamine release into the insular cortex, but CA1 hippocampal dopamine remains unaltered during the sample phase (Guzmán-Ramos et al., 2012). Significantly, hippocampal catecholaminergic denervation by 6-OHDA administration impedes OLRM but spares NOR (Moreno-Castilla et al., 2017), indicating that the hippocampal dopaminergic activity is not involved in NOR formation or retrieval. Hence, the fine modulation of the dopaminergic system is required to establish recognition memory appropriately.

The inactivation of VTA (Rossato et al., 2013) or the denervation of the mesolimbic-cortical dopaminergic terminals (Stephen Fink and Smith, 1980) hinders NOR consolidation. While an excess of dopaminergic levels, caused by knocking out the expression of the dopamine transporter, impedes NOR formation (Chang et al., 2020). Similarly, systemic administration of methamphetamine, an enhancer of catecholamines release (Belcher et al., 2008; Camarasa et al., 2010), or the blockade of D2 receptors or D4 receptors, impairs NOR establishment (Besheer et al., 1999; Woolley et al., 2003; Watson et al., 2012; Miyauchi et al., 2017). Moreover, the systemic activation of D1-like receptors hinders OLRM and NOR retrieval (Hotte et al., 2005; Pezze et al., 2015), while the inactivation of D3 (Watson et al., 2012) enhances novel recognition retrieval. Likewise, memory persistence and memory retrieval in NOR are heightened after the systemic administration of a D1/D5 receptor agonist (Hotte et al., 2005, 2006; de Lima et al., 2011; see Table 1). Regarding the hippocampus, administering a D1 antagonist after the sample phase into the dentate gyrus impairs object recognition (Yang et al., 2017). However, administering a D1/D5 receptor antagonist into the dorsal hippocampus before or after the sample phase spares long-term NOR memory (Balderas et al., 2013a; Rossato et al., 2013). Conversely, the administration of a D1/D5 receptor antagonist into the perirhinal cortex before the sample phase spares short-term but prevents the consolidation of NOR (Balderas et al., 2013a). Moreover, the intracerebroventricular administration of D1-like receptors antagonist impairs spatial novel configuration learning (Lemon and Manahan-Vaughan, 2006), whereas blockade of D1 receptors within the prefrontal cortex or amygdala (Nagai et al., 2007; Rossato et al., 2013) impairs NOR consolidation (see Table 2).

**Table 1:** Systemic pharmacological effects of catecholaminergic drugs on NOR and OLRM performance.

	Drug	Mechanism	Time of administration	Task	Effect on memory	References
α and β						
	Epinephrine	Agonist	Post-training	NOR	↑	Dornelles et al. (2007)
α						
	Yohimbine	Agonist	Post-training	NOR	↑	Roozendaal et al. (2007) and Song et al. (2021)
	Yohimbine	Agonist	Post-training	OLRM	↑	Song et al. (2021)
Dopamine uptake transporter						
	Methamphetamine	Stimulates release of dopamine	Chronic, one week before training	NOR	↓	Belcher et al. (2008) and Camarasa et al. (2010)
D2/D3						
	Eticlopride / Raclopride	Antagonist	Before retrieval/Before training	NOR	↓	Besheer et al. (1999) and Woolley et al. (2003)
D1/D5						
	SCH-23390	Antagonist	Before retrieval	NOR	↓	Besheer et al. (1999)
	SKF81297	Agonist	Before retrieval	OLRM	↓	Hotte et al. (2005)
	SKF81297	Agonist	Before retrieval	NOR	↓	Hotte et al. (2005)
	SKF38393 / SKF81297	Agonist	Post- training/Before retrieval	NOR	↑	Hotte et al. (2005); Hotte et al. (2006) and de Lima et al. (2011)
D2						
	PD128, 907	Antagonist	Before retrieval	NOR	↓	Watson et al. (2012)
D3						
	S33084	Antagonist	Before retrieval	NOR	↑	Watson et al. (2012)
D4						
	L-745, 870	Antagonist	Before training	NOR	↓	Miyauchi et al. (2017)

*↑ indicates enhanced memory and ↓ indicates impaired memory*.

**Table 2:** Pharmacological effects of catecholaminergic drugs on NOR and OLRM performance.

	Receptor	Drug	Mechanism	Time of administration	Task	Effect on memory	References
Intracerebroventricular		
	D1/D5	SCH-3390	Antagonist	Before training	Object-in-context	↓	Lemon and Manahan-Vaughan (2006)
Anterolateral hypothalamus						
	Dopamine and noradrenaline uptake transporter	6-OHDA	Neuotoxin	7 days before training	NOR	↓	Stephen Fink and Smith (1980)
Basolateral amygdala					
	β	Propranolol	Antagonist	Post-training	Object-in-context	↓	Barsegyan et al. (2014)
	α and β	Norepinephrine	Agonist	Post-training	Object-in-context	↑	Barsegyan et al. (2014)
	D1/D5	SCH-23390	Antagonist	Post-training	NOR	↓	Rossato et al. (2013)
Dorsal hippocampus					
	Dopamine and noradrenaline uptake transporter	6-OHDA	Neuotoxin	7 days before training	NOR	=	Moreno-Castilla et al. (2017)
	Dopamine and noradrenaline uptake transporter	6-OHDA	Neuotoxin	7 days before training	OLRM	↓	Moreno-Castilla et al. (2017)
	β	Timolol	Agonist	Post-training	NOR	↑	Mello-Carpes et al. (2016)
	α and β	Norepinephrine	Agonist	Post-training	NOR	↑	Mello-Carpes et al. (2016)
	D1/D5	SCH3390	Antagonist	After training	NOR	↓	Yang et al. (2017)
	D1/D5	SCH-23390	Antagonist	Post-training	NOR	=	Rossato et al. (2013)
	D1/D5	SCH-23390	Antagonist	Before training	NOR	=	Balderas et al. (2013a)
	D1/D5	SKF38393	Agonist	Before training	NOR	=	Balderas et al. (2013a)
Medial Prefrontal cortex					
	D1/D5	SCH-23390	Antagonist	Post-training	NOR	↓	Rossato et al. (2013)
Nucleus accumbens (Core or shell region)			
	Dopamine and noradrenaline uptake transporter	6-OHDA	Neuotoxin	7 days before training	OLRM	↓	Nelson et al. (2010)
Nucleus accumbens (Core region)			
	Dopamine and noradrenaline uptake transporter	6-OHDA	Neuotoxin	7 days before training	NOR	=	Nelson et al. (2010)
Perirhinal cortex			
	D1/D5	SCH23390	Antagonist	Before training	NOR	↓	Balderas et al. (2013a)
	D1/D5	SKF38393	Agonist	Before training	NOR	↑	Balderas et al. (2013a)
Prefrontal cortex			
	D1/D5	SKF81297	Agonist	Before training	NOR	↓	Pezze et al. (2015)
	D1	SCH-23390	Antagonist	Before training	NOR	↓	Nagai et al. (2007) and Rossato et al. (2013)
	D2	Raclopride	Antagonist	Before training	NOR	=	Nagai et al. (2007), Rossato et al. (2013), and Pezze et al. (2015)
VTA		
	GABA A	Muscimol	Agonist	Post-training	NOR	↓	Rossato et al. (2013)

*↑ indicates enhanced memory, ↓ indicates impaired memory and = indicates spared memory*.

All these results suggest that catecholaminergic activity, particularly dopaminergic modulation, is responsible for enhancing the consolidation of recognition memory. Exposure to novel stimuli induces dopamine release in several brain structures, facilitating memory establishment through the synthesis, tagging, and capture of proteins associated with synaptic plasticity generated by learning signals (Frey and Morris, 1998). In the absence of a neuromodulator, modified synapses return to the baseline level after learning, reducing the probability of consolidating memories (Takeuchi et al., 2016; Duszkiewicz et al., 2019). Therefore, it has been suggested that dopamine modulates learning signals that facilitate the consolidation of events (Montague et al., 2004) involved in the process of familiarity consolidation. Thus, dopamine might modulate perceptual salience consolidation signals, enabling recognition memory.

## Dopamine and Perceptual Salience

As previously reviewed, dopaminergic signaling is widely associated with neuronal plasticity enhancement. In general, dopamine activates D1-like and D2-like receptors triggering a cascade of events that lead to cellular modifications and the induction of protein synthesis necessary for memory consolidation. Therefore, dopaminergic activity is related to the modulatory effect of object and location recognition memory establishment. Although dopaminergic activity contributes significantly to memory processes, it remains to elucidate the precise functional role of dopamine and its specific contribution to perceptual salience processing necessary for recognition memory evaluated through NOR and OLRM tasks. Novelty-related dopaminergic activity within several brain structures is involved in recognition memory. Dopamine release has been related to motivated behaviors and predicting and coding rewarding events (Schultz et al., 1993; Schultz, 1998). The evidence shows that VTA dopaminergic neurons increase their firing rate to signal reward (Berridge and Robinson, 1998; Nomoto et al., 2010; Fiorillo, 2013). Nevertheless, evidence exhibits that exposure to aversive stimuli also increases the electrical activity within VTA (Brischoux et al., 2009; Bromberg-Martin et al., 2010). Whereas unexpected stimuli prediction errors also modulate dopaminergic activity (Schultz, 1998). Therefore, novelty, the intrinsic value of the stimulus (valence), and unforeseen modifications in predicted events induce changes in dopaminergic response, probably on behalf of salience (Horvitz, 2000).

Consequently, exposure to novel stimuli triggers dopamine release facilitating memory consolidation (Balderas et al., 2013a; Osorio-Gómez et al., 2021). Notably, salient visual stimulation induces a short-latency electrical response in the substantia nigra (Comoli et al., 2003). This phasic dopaminergic activity is strongly related to salience (Bromberg-Martin et al., 2010; Barto et al., 2013; Cho et al., 2017). Intrinsically salient stimuli compete for attention, in which new and relevant visual stimuli drive attentional processes (Yantis and Hillstrom, 1994). Novelty-induced salience influences memories, attention, and motivation through dopaminergic activity (Puglisi-Allegra and Ventura, 2012). Salient stimuli prioritize the consolidation of the relevant over neutral information (Alger et al., 2019). Interestingly, it has been suggested that the brain is organized to promote the interaction of functional networks, including the salience network (Tsai et al., 2020). Evidence indicates that the insular cortex is a crucial node of the salience network (Uddin, 2015), integrating exteroceptive and interoceptive information (Seeley et al., 2007). The insular cortex might be involved in salience because of the multiple inputs arising from the amygdala, VTA, the dorsomedial nucleus of the thalamus, and the prefrontal cortex (Bermudez-Rattoni, 2014; Uddin, 2015; Gil-Lievana et al., 2020, 2022; Chen et al., 2022).

**Figure 2:**
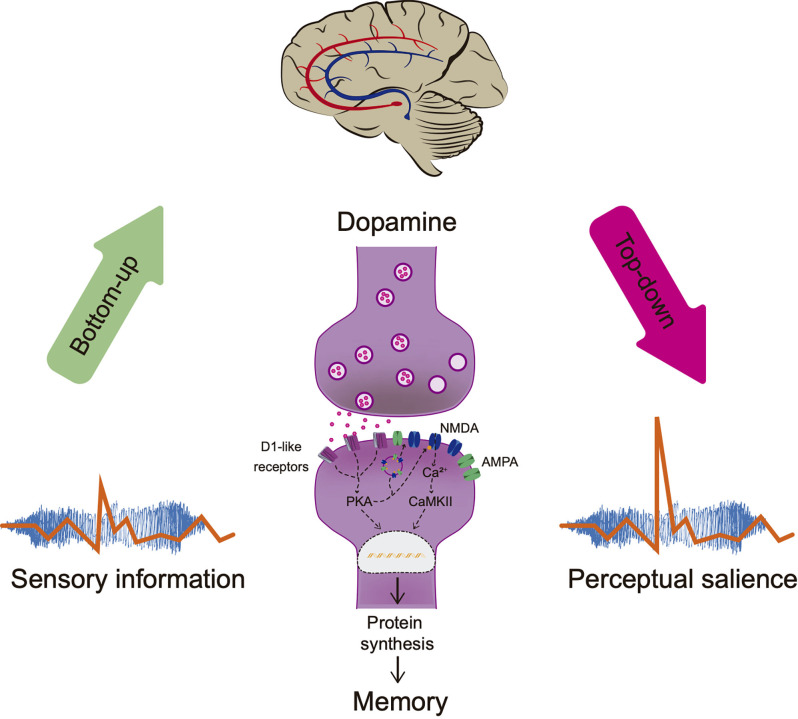
Perceptual salience. Sensorial stimulation attracts attentional resources bottom-up facilitating information processing. Brain hierarchizes salient information according to the physical stimuli properties and the organism’s experience (meaning, consequences, or relationship with other stimuli in the environment). Dopamine regulates neuronal plasticity through activation of D1-like receptors enhancing memory consolidation, improving NMDA’s conductance, inducing protein synthesis, and AMPA/NMDA receptors externalization. Memory modulates top-down processing without altering the initial sensory perception necessary for recognition by comparing the stimuli with the stored information and adjusting salience processing, a term referred to as perceptual salience. PKA, Protein Kinase A; Ca^2+^, calcium; CaMKII, calcium calmodulin kinase.

As mentioned, the physical properties of the stimulus drive cognitive processes contrasting it with the surroundings and attracting attentional resources facilitating information processing. However, the physical properties of the stimuli are not the only components that can direct information processing. Previous experiences modulate top-down processing, by which “internal” factors and cognition influence perception (Awh et al., 2012). Thus, information is hierarchized within the brain according to the physical properties of the stimulus or to the previously learned information related to that stimulus, facilitating the perception of the stimuli in future events. Consequently, perceptual salience requires integrating information into meaningful-related memories without changing the initial detection of the stimuli (see Figure 2).

The hippocampus is involved in detecting salient spatial stimuli; hippocampal formation increases its activity when new contextual information is presented. This novelty signal is transferred to the VTA and contributes to the activation pattern observed in the VTA during novelty seeking (Lisman and Grace, 2005). For instance, the temporal inactivation of the VTA impairs NOR consolidation (Rossato et al., 2013), indicating that the dopaminergic system is essential for object recognition consolidation. This information suggests that the hippocampus and the dopaminergic inputs from the VTA and the LC form a functional loop that detects novelty and compares this information to previously integrated memories. Thus, dopaminergic activity might control the entry of crucial adaptative information and promotes subsequent integration into long-term memory through modification of synaptic plasticity (Lisman and Grace, 2005; Lisman et al., 2011).

There are illustrative examples of perceptual salience modulation *via* dopaminergic regulation. Optogenetic stimulation of the dopaminergic neurons within the VTA enhances behavioral response (taste neophobia) to the low-intensity stimulus facilitating taste recognition memory consolidation (Gil-Lievana et al., 2022). This effect was probably to bottom-up salience enhancement since augmented behavioral responses were observed during stimulation (acquisition). However, optogenetic stimulation of dopaminergic terminals from the VTA to the insular cortex does not modify the behavioral response (lack of neophobia) to the subthreshold stimuli while facilitating taste recognition memory performance during retrieval. It is essential to mention that optogenetic activation of VTA neurons or terminals does not alter the valence of the stimulus (Gil-Lievana et al., 2022), suggesting an increased effect over memory consolidation despite the low salient stimulus. Consequently, dopaminergic stimulation within the insular cortex enhances the perceptual salience for low-intensity stimulus. Similar results are observed after the optogenetic stimulation of LC-dopaminergic inputs into the hippocampus, which evokes dopamine release and enhances OLRM consolidation (Kempadoo et al., 2016).

Additionally, perceptual salience enhancement requires activation of the D1-like receptor. The blockade of cortical D1 receptors impairs long-term recognition memory keeping the short-term recognition memory intact. Conversely, administration of a D1 receptors agonist into the perirhinal cortex (SKF38393) before a subthreshold stimulation in a NOR protocol does not increase exploration times during acquisition. However, it induces NOR consolidation enhancement causing a clear novel object discrimination during the test (Balderas et al., 2013a). Although the blockade of D1-like receptors within the dorsal hippocampus spares NOR consolidation (Balderas et al., 2013a; Rossato et al., 2013). Recent studies report that the administration of dopamine or a D1 receptor agonist into the dorsal hippocampus enhances NOR persistence (Vargas et al., 2020; Lima et al., 2022). These results suggest that the memory trace formation expressed in short-term memory is not dopamine-dependent, but its activity enables long-term and persistent storage (Balderas et al., 2013a; Moreno-Castilla et al., 2017; Vargas et al., 2020). In contrast, the blockade of D1-like receptor activity within the insular cortex impedes perceptual salience enhancement (Gil-Lievana et al., 2022). Hence, dopaminergic stimulation promotes the consolidation and persistence of stimuli that under normal conditions are not possible considering their subthreshold properties. In this regard, it has been suggested that dopaminergic responses are not related to signaling the stimuli’s intensity but rather to the perceived intensity (de Lafuente and Romo, 2011). Although, these authors concluded that midbrain dopamine neurons code the subjective perception of the event (Romo and Rossi-Pool, 2020). We suggest that dopaminergic activity within the hippocampus, perirhinal and insular cortices facilitate the consolidation of information into long-term and persistent memories enabling the perceptual salience to guide behavior efficiently and effectively in future encounters. Considering that dopamine modulates synaptic plasticity, dopaminergic signaling promotes the consolidation of inconspicuous stimulus into a relevant memory without altering the initial sensory perception.

## Impact of Catecholaminergic Alterations in Recognition Memory

Dysregulation of dopaminergic signaling hinders declarative memories such as recognition memory (Guzmán-Ramos et al., 2012; Moreno-Castilla et al., 2017; Hernández-Ramírez et al., 2021). In some pathological brain conditions, such as schizophrenia, Alzheimer’s, and Parkinson’s diseases, the alteration in the catecholaminergic system leads to cognitive impairments. Schizophrenia is a mental disorder characterized by psychotic events that alter beliefs, perceptions, and emotions (Kapur, 2003). It has been demonstrated that over-activation of the dopaminergic pathways is associated with schizophrenia (Brisch et al., 2014; Winton-Brown et al., 2014). Consequently, dysregulated dopamine transmission leads to a stimulus-independent release of dopamine, generating an aberrant assignment of salience to external objects, exaggerating the perception (Kapur, 2003). Thus, aberrant perception salience involves attentional resources during irrelevant stimuli coding and drives cognitive processes inappropriately (Roiser et al., 2013). This distorted perception results from excess dopamine signaling within several brain areas, such as the ventral striatum, the prefrontal cortex. and the hippocampus (please see Kapur, 2003; Roiser et al., 2013). Noticeably, dopaminergic alterations in functional connectivity have been reported in the salience network, including dysfunctional connectivity in the anterior insular and anterior cingulate cortices, which correlates with excessive salience attributable to internal experiences (Rössler et al., 2020). In animal studies, systemic administration of methamphetamine hinders NOR due to dopaminergic system over-activation (Belcher et al., 2008; Herring et al., 2008; Camarasa et al., 2010; Razavi et al., 2020; Khodamoradi et al., 2022). Additionally, the dopamine transporter knockout mouse mimics specific symptoms observed in schizophrenia due to the increased dopaminergic activity; this mouse model also exhibits memory impairments, including NOR alterations (Wong et al., 2012). Most antipsychotic pharmacological treatments involve dopaminergic regulation. These drugs alleviate schizophrenia symptoms and ameliorate NOR deficits, the systemic administration of a D4 receptor agonist (Miyauchi et al., 2017), a D3 (Sun et al., 2016; Gou et al., 2017), and a D2 antagonist (McIntosh et al., 2013) improves the recognition for novel objects in animal models of schizophrenia mental disorder.

Alzheimer’s disease is a progressive neurodegenerative disorder distinguished by the accumulation of amyloid β oligomers, plaques, and neurofibrillary tangles. Recent studies have suggested that the catecholaminergic system is affected during the first stages of the pathology. The neurodegeneration initiates within the LC (Braak and Del Tredici, 2015) and the VTA (Serra et al., 2018), propagating to the medial temporal lobe and cortical regions (Flores et al., 2022; Guzmán-Ramos et al., 2022), causing cognitive impairments. Our group reported that the accumulation of β-amyloid in a transgenic mouse model of Alzheimer’s disease induces catecholaminergic neuronal loss (Moreno-Castilla et al., 2016). Importantly, in the same mouse model, animals exhibit NOR and OLRM impairments; these effects are attributable to a failure in dopamine release (Guzmán-Ramos et al., 2012; Moreno-Castilla et al., 2017). Moreover, stimulation of the dopaminergic system through the systemic administration of dopamine precursor levodopa (Ambrée et al., 2009) or a dopamine reuptake blocker (Guzmán-Ramos et al., 2012) attenuates NOR impairment observed in Alzheimer’s disease mice models. Although memory deficits are strongly related to Alzheimer’s disease, some patients exhibit sensorial alterations, including visual, olfactory, somatosensory, and auditory impairments (Mapstone et al., 2006; Daulatzai, 2016), probably due to early catecholaminergic alterations (Rey et al., 2012).

Parkinson’s disease is another progressive neurodegenerative disorder related to the dysfunction of the catecholaminergic system. This pathology is characterized by several motor symptoms caused by aggressive dopaminergic cell loss in the substantia nigra (Lotharius and Brundin, 2002). Furthermore, Parkinson’s disease patients also exhibit cognitive detriments (Aarsland, 2016). Administration of 1-methyl-4-phenyl-1,2,3,6-tetrahydropyridine, a selective dopaminergic cell neurotoxin, into the substantia nigra is widely used as an animal model for Parkinson’s disease; rats treated with this neurotoxin display degeneration of nigrostriatal dopaminergic neurons and NOR impairment (Sy et al., 2010; Chang and Wang, 2021). The administration of 6-OHDA into the striatum generates NOR (Chao et al., 2013; Masini et al., 2018) and OLRM deficits (Xie and Prasad, 2020; Barón-Quiroz et al., 2021). Moreover, the chronic treatment of reserpine, a monoamine-depleting agent, is also employed as a pharmacological model of Parkinson’s disease in rodents; this animal model likewise shows long-term NOR memory impairment (Ikram and Haleem, 2019). A transgenic mice model of Parkinson’s disease (MitoPark) resembles progressive neurodegeneration and death of dopaminergic neurons, loss of motor function, and deficits in NOR tasks (Li et al., 2013). This evidence shows that NOR and OLRM memory decline accompanies dopaminergic dysfunction in Parkinson’s disease models. Although these Parkinson’s disease models are suitable for emulating motor alterations, it is essential to mention that cognitive deficits in NOR and OLRM tasks appear before motor symptoms. Recognition memory impairments observed in Parkinson’s disease could also be explained due to alterations in perception, visual hallucinations being the most frequently observed in Parkinson’s patients (for a review, Russo et al., 2019); probably caused by alterations in the dopaminergic system.

Therefore, deficits in NOR and OLRM task performance exhibited in many animal models of brain disorders result from dopaminergic dysregulation. Catecholaminergic alterations reported as hypoactivation or hyperactivation disrupt the finely tuned dopaminergic system, probably negatively impacting the salience circuits involved in recognition memory. Thus, Alzheimer’s, Parkinson’s, schizophrenia, and other brain disorders related to altered catecholaminergic signaling might alter the integration of information required for perceptual salience and the subsequent cognitive processes such as memory, attention, and motivation.

## Conclusions

Relevant information is more efficiently processed in comparison to non-relevant stimuli. Selection of relevant information from the environment is accomplished since intense physical properties are intrinsically salient. However, salience could also be modulated by “internal” factors according to the observer’s experience. Hence, perceptual processing occurs according to the intense physical properties of the stimulus or the previously learned information. Former perceptual processing requires a close interaction between perception and memory, considering that integrated memories modulate perception and salience processing. Here, we propose the term perceptual salience to explain how memory mediates the integration of inconspicuous stimuli into a relevant memory trace, facilitating salience detection in future encounters without apparently altering the recognition of the physical attributes or valence of the stimuli.

Memory is a fundamental cognitive function that integrates information allowing recognition of familiar (non-salient) events from novel (salient) ones. In general, recognition memory involves acquiring, consolidating, retrieving, and updating two differential processes: recollection and familiarity. Recollection recovers the characteristics of the stimulus within a context, whereas familiarity integrates whether a stimulus is new or has already been experienced. Several brain regions integrate new information learning; the hippocampus, prefrontal cortex, parahippocampal (perirhinal, entorhinal, and postrhinal), and insular cortices are widely involved in recognition memory. Moreover, the catecholaminergic system modulates cognitive functions, including recognition memory. The main catecholaminergic inputs arise from the LC and the VTA, which innervate several brain structures related to recognition memory. Novel object and object location recognition memory are the most common behavioral paradigms employed to determine recognition memory’s acquisition, consolidation, retrieval, and updating due to the natural tendency of rodents to explore novel stimuli. Exposure to novel stimuli or spatial configuration induces a dopamine release, modulating dopaminergic receptors that strengthen learning signals, and facilitating the transition of novelty to familiarity. Mainly, dopaminergic activity within the perirhinal and insular cortices and the hippocampus mediates the consolidation process of perceptual salience. Dopamine regulates plasticity-related events that enhance memory consolidation and persistence, regardless of the initial sensory perception, improving perceptual salience during recognition memory. Importantly, brain disorders caused by neurodegenerative diseases such as Alzheimer’s or Parkinson’s, metabolic disorders, or schizophrenia alter recognition memory due to dopaminergic dysfunction, probably related to the distortion in perceptual salience and the subsequent cognition processes.

## Author Contributions

The authors confirm contribution to the manuscript as follows: DO-G, KG-R, and FB-R conceptualized, reviewed, and edited the manuscript; preparation of draft manuscript by DO-G. All authors contributed to the article and approved the submitted version.

## Conflict of Interest

The authors declare that the research was conducted in the absence of any commercial or financial relationships that could be construed as a potential conflict of interest.

## Publisher’s Note

All claims expressed in this article are solely those of the authors and do not necessarily represent those of their affiliated organizations, or those of the publisher, the editors and the reviewers. Any product that may be evaluated in this article, or claim that may be made by its manufacturer, is not guaranteed or endorsed by the publisher.
